# Perinatal Outcome in Assisted Reproductive Pregnancies: Comparative Analysis of Reduced versus Unreduced Gestation

**DOI:** 10.1155/2016/7504609

**Published:** 2016-11-23

**Authors:** Shilpa Bhandari, Pallavi Agrawal, Ishita Ganguly, Aparna Singh, Nitika Gupta

**Affiliations:** Department of Reproductive Medicine and Surgery, Sri Aurobindo Medical College and Post Graduate Institute, Indore, Madhya Pradesh, India

## Abstract

*Objectives*. This study aims to evaluate perinatal outcomes such as gestational age at delivery and live birth rate in singleton and twin gestation with or without fetal reduction.* Method*. A retrospective analysis was done on patients which were divided into reduced and unreduced groups on the basis of order of reduction of one or more fetuses between 6 and 13 weeks of gestation. Patients records were studied to note gestational age at delivery/abortion, birth weight, and neonatal outcome.* Result*. The cohort included a total of 292 patients: 102 singletons and 190 twins. 52 pregnancies were reduced in singleton cohort and 68 were reduced in twin cohort. No statistical difference was observed in live birth rate, gestational age at delivery, and birth weight and significant higher incidence of IUGR was observed in reduced and unreduced twin gestation. In singleton pregnancies however preterm delivery rate increased with fetal reduction.* Conclusion*. Although reduction does not reduce the live birth rate, it does reduce gestation age of delivery and birth weight of newborn. This effect is more apparent when multiple gestation is reduced to singleton.

## 1. Introduction

In this era of exponential industrialization and global changes, lifestyle disorders like infertility are on the rise. It is estimated that infertility affects 8 to 12 per cent of couples worldwide [1]. In India, the overall prevalence of primary infertility varies from 3.9 to 16.8 per cent [2].

The need for single embryo transfer has been highlighted in recent studies globally but it is limited because of several obstacles. The complications of multiple gestation (increase in abortion, preterm delivery, maternal morbidity, etc.) are often ignored by both the couple and the physician against the instant gratification provided by increase in pregnancy rate.

At the end when primary preventive strategies to prevent multiple pregnancies have failed or circumstantial considerations have warranted transfer of higher number of embryos resulting in multiple gestation, fetal reduction is an interventional step to be considered. As stated in 2006 by the International Federation of Gynecologists and Obstetricians Ethics Committee Report, “multiple pregnancy of an order of magnitude higher than twins involves great danger for the woman's health and also for her fetuses, which are likely to be delivered prematurely with a high risk of either dying or suffering damage,” and “where such pregnancies arise, it may be considered ethically preferable to reduce the number of fetuses rather than to do nothing” [3].

Taking into account the legitimacy of the procedure, a related concern is whether fetal reduction to twins further compromises the outcome of the reduced pregnancy as compared with a twin pregnancy that had not undergone such a procedure. There has been dilemma over the advantage of fetal reduction due to contrasting results by various studies [4–6].

Since perinatal outcome may vary with regional factors like level of medical care and patient profile, this study helps bring forth the answers to these questions in the context of central India. Therefore the aim of this study was to compare perinatal outcome of pregnancies conceived through ART—singleton versus twin gestation. Effects of reduction in multiple pregnancies by comparing reduced singleton and twins with their unreduced counterparts were also analysed.

## 2. Material and Methods

This retrospective cohort study was performed in the Department of Reproductive Medicine and Surgery in Sri Aurobindo Medical College and Post Graduate Institute during January 2012 and December 2015. All patients who conceived by in vitro fertilization (IVF) or intracytoplasmic sperm injection (ICSI) were included in the study. Only patients who had aborted or delivered during the study period were included. Details of pregnancies were recorded in the departmental record in the form of individual patient file which was periodically updated either at time of patient follow-up visit or by telephonic enquiry.

Patients, who were lost to follow-up, had incomplete records, had heterotopic pregnancies, had monochorionic pregnancies, or had decided to continue triplet or higher order gestation, were excluded from the final evaluation. Patients who aborted all the fetuses before 13 weeks spontaneously or following MFPR were also excluded from the final evaluation.

As per departmental protocol, ultrasonography (USG) was initially performed to determine the location and number of gestational sacs when quantitative *β*-HCG levels were expected to be 2000 mIU/mL or more between 5.5 and 6.5 gestational weeks (3.5 to 4.5 weeks after embryo transfer). USG was repeated every 2 weeks until the 12th gestational week, transvaginally. Pregnant women were followed up in the department under a fetal medicine specialist till 12 weeks. Patients presenting to local obstetrician were followed up periodically on telephone.

In patients with triplet or quadruplet gestation multifetal pregnancy reduction (MFPR) at 11–13 weeks was done such that 2 fetuses were reduced in quadruplet pregnancies and single fetus was reduced in triplet pregnancies. Fetal reduction was performed transabdominally under USG guidance after establishing chronicity and nuchal translucency. Any fetus who appears abnormal or had increased nuchal translucency was chosen for fetal reduction. Otherwise in all normal looking fetuses one who was located near fundus was chosen for reduction. The period of 11–13 weeks was chosen for the MFPR for selecting the fetus with increased nuchal translucency for reduction. The procedure was performed by injection of approximately 2 to 3 mL of potassium chloride (concentration 2 mEq KCl/mL) into the fetal thorax using an 18-gauge spinal needle by a single operator.

Two study groups were defined on the basis of USG findings at 13 weeks.

Group A: USG showing single live fetus were defined as singleton gestation.
Group B: USG showing twins gestation were defined as twins gestation.

These groups were further subdivided into reduced and nonreduced group.

Fetal reduction (FR) was defined as disappearance of gestational sac or loss of cardiac activity in one or two gestational sacs (after its identification). Patients who lost one or more fetuses spontaneously before or after MFPR (but before 13 weeks) were included in reduced group. Fetal reduction could have occurred spontaneously or iatrogenically due to induced fetal reduction procedure.

Nonreduced group: a number of live fetuses at six weeks were similar to those seen at 13 weeks.

In the reduced pregnancy group note was made according to the number of fetuses reduced (4 to 2, 4 to 1, 3 to 2, and 3 to 1).

For each pregnancy included, the following data were retrieved from the medical record: maternal age, parity, fetal reduction procedure, gestational age (in weeks) at abortion/delivery, birth weights, and number of live-born infants.

Late abortion was defined as disappearance of cardiac activity in utero or delivery before 28 completed weeks of gestation. Preterm delivery was defined as birth of a viable baby (after 28 weeks) at or before 37 completed weeks of gestation. Very preterm delivery was defined as birth of a viable baby between 28 to 32 weeks of gestation. Neonatal death was defined as death of a live baby within 4 weeks of delivery. Restricted fetal growth or IUGR was defined as a birth weight less than the 10th percentile for gestational age on the basis of national singleton birth weights [7].

The outcome variables studied in the present study were pregnancy loss, weeks of gestation at delivery, birth weight of the baby, and incidence of IUGR.

Measurement data underwent normality test and were expressed as mean ± standard deviation. The maternal and fetal parameters of the two groups were compared using chi-square test and *t*-test wherever applicable to determine statistical significance. Association of number of fetuses with perinatal outcome was evaluated using chi-square test.

One-way analysis of variance (ANOVA) was used to calculate the significant difference in mean gestational age at delivery and birth weight in different subgroups (4 to 2, 4 to 1, 3 to 2, and 3 to 1). Statistical significance was established at *p* < 0.05.

## 3. Results

A total of 292 patients were included in the study. Out of 292 patients 102 patients had single and 190 patients had twin gestation at 13 weeks of pregnancy on the basis of number of fetuses seen in transvaginal scan.

52 pregnancies (6 from triplets to singleton and 46 from twins to singleton) were reduced to singleton either spontaneously or iatrogenically in singleton cohort whereas 68 pregnancies (11 from quadruplets to twins and 57 from triplets to twins) were reduced to twins in twin cohort.

In singleton group, with multigravida patients out of 13 only 4 patients had previous conception that crossed the period of viability. In twins group out of 16 multigravida patients only 5 patients had previous conception that had crossed the period of viability.

In both singleton and twin groups, there was no statistical difference between the average age of patients who underwent fetal reduction versus those who did not (*p* = 0.1881 and 0.983, resp.) (Table 1). Prepregnancy BMI was also similar in both groups.

The percentage of patients who had live birth also is similar in reduced and unreduced pregnancy in both singleton and twin gestation (*p* = 0.259 and 0.832, resp.). However unreduced singleton pregnancies had significantly higher chance of term delivery in comparison to those who underwent fetal reduction (*p* = 0.0421). Similar comparison in twin gestation did not show any statistical significance.

IUGR was more common in twin reduced pregnancy as compared to twin unreduced pregnancy. However, neonatal death was similar in both reduced and unreduced pregnancies in both singleton and twin groups.

In singleton pregnancies, the 102 patients were divided according to the number of gestational sacs seen at 6 weeks and 13 weeks into 3 to 1, 2 to 1, and 1 subgroups. In 2 to 1 subgroup all patients were reduced spontaneously into singleton pregnancy and did not undergo any iatrogenic fetal reduction, whereas, in 3 to 1 subgroup, 5 patients were reduced from triplets to twins iatrogenically at 12 weeks and all reduced to singleton spontaneously at 13 weeks. Another 1 patient in 3 to 1 subgroup was reduced to singleton spontaneously. One only subgroup had single pregnancy at 6 weeks and no fetal loss was seen at 13 weeks. When the gestational age at delivery was compared in live births of this group we found a significant association with the number of fetuses reduced. When no fetal reduction occurred, maximum patients delivered at term (*p* = 0.03). A similar pattern was noted in terms of birth weight too. A statistically significant association was seen with no fetal reduction and higher birth weight in singleton gestation (*p* = 0.012) (Table 2).

In the present study 190 patients with twin gestation delivered 394 babies. The patients were divided into 3 subgroups. Subgroup (4 to 2) was those in whom quadruplet gestation at 6 weeks was reduced to twins by 13 weeks (MFPR or SPR). Similarly 3 to 2 formed another subgroup while patients who conceived twin gestation and continued without any fetal loss till 13 weeks were put in the last subgroup. In 4 to 2 subgroups 4 pregnancies and in 3 to 2 subgroups 16 pregnancies were reduced spontaneously.

We did not find any statistical association between the three groups in terms of abortion/live birth rate (*p* = 0.874), gestational age at delivery (*p* = 0.572), and birth weight (*p* = 0.566) (Table 3). In patients who did not undergo any reduction, higher birth weight at delivery was noted in comparison to the other two subgroups although it was not statistically significant (Table 3).

A total of 394 babies were delivered at or later than 28 weeks (period of viability) as singleton or twin pregnancies. The chance of higher birth weight was significantly more in patients where fewer fetuses were reduced. In singleton pregnancies, which did not undergo fetal reduction, the babies had significantly higher birth weight. Overall 40.1% babies had birth weight above 2.6 kg and 66.23% (51/77) of them were singleton pregnancies at 13 weeks. In cases of twin deliveries, 26 babies have birth weight above 2.6 kg. Out of these 65.38% (17/26) did not undergo reduction (Table 4).

## 4. Discussion

Even though elective single embryo transfer is ideal in certain circumstances, as in repeated failure cases, we sometimes deliberately transfer a large cohort of embryos, thus taking a thoughtful risk for a high order gestation. In other cases, owing to a limited ability to select the embryos with the highest chance for implantation, we may also choose the introduction of multifetal transfer, knowing our patient may end up with a multifetal pregnancy.

### 4.1. Age and Parity

We have found no association of age and previous parity on the perinatal outcomes in any groups—reduced singleton/twins and unreduced singleton/twins. This is in contrast to many studies done in past which consider increasing maternal age [5] and nulliparity [8] to be associated with preterm delivery and abortions. It is possible that nulliparous patients in these studies had a subpopulation, which was older, with some associated uterine factor, as well as poorer oocyte quality, which led the whole group towards lower performance.

### 4.2. Effect of Reduction on Abortion Rate

This study demonstrates no clinical difference in abortion rate in reduced and unreduced pregnancy (both singleton and twin gestation). In fact, it was little higher in the unreduced twin pregnancies. Similar results were seen in previous study also [9]. This not just reflects a lack of causal relationship between fetal reduction and abortion but also reinforces the need to counsel the patient regarding the possibility of pregnancy loss in all multiple gestation irrespective of reduction. In our study least percentage of abortions were noted in the unreduced singleton group. However the comparison between multiple gestation reduced to singleton (3 to 1 and 2 to 1) and singleton itself did not show any statistical significance. The analysis of this may have been hindered by absence of higher numbers in the reduced singleton group. Therefore we may conclude that though reduction in itself does not seem to predispose to abortion as such, reduction to singleton pregnancy purposefully is not worthy of advocacy.

### 4.3. Outcome of Singleton Pregnancies

Present study shows that singleton gestation has better perinatal outcomes than twin gestation. Pregnancies that had undergone reduction to singleton also fared better than those reduced to twins though these differences did not reach statistical significance in terms of average gestational age at delivery and live birth rate. However lower birth weight and preterm deliveries in singleton pregnancies were significantly associated with those who had undergone reduction. It is pertinent to point out here that reduced singleton birth weight though lower than unreduced singleton was significantly higher than reduced twin gestation.

It is important to mention that some previous authors [10] have found singleton pregnancies of ART to be at higher risk than those conceived spontaneously. It makes us wonder if ART in itself predisposes to worsening of perinatal outcomes and increase in gestational sacs merely compounds the problem. In other words an added risk, such as assisted conception, may have a marked impact on a low risk singleton pregnancy but only a small effect on the heavily weighted balance of twin pregnancy.

### 4.4. Outcome of Twin Gestation

In a prospective study [4] which compared reduced with unreduced twin gestation, preterm delivery rate was similar in unreduced twins. The gestational ages in both groups were found to be similar at around 35 weeks. In present study the average gestational age at delivery is similar (35 weeks) in reduced and unreduced group. We did not find any significance even when we analysed the reduced group according to the number of fetuses reduced (4 to 2 and 3 to 2). We too found a slight (though nonsignificant) increase in abortions and preterm deliveries in twins without any reduction. In an older study [11] fetuses reduced to twins from triplets showed abortion and preterm delivery rates similar to unreduced twins. It is possible that subtle patient factor especially higher age and lower parity in unreduced twin group may be responsible for the slight differences in findings.

The incidence of IUGR was higher in reduced twins as compared to unreduced twins. Similar to our study Depp et al. [12] reported the incidence of intrauterine growth restriction as 19.4% in the nonreduced twins, 36.3% in pregnancies reduced from triplets, and 41.6% in pregnancies reduced from quadruplets.

### 4.5. Comparison between Twin and Singleton Pregnancies

Gleicher et al. [13, 14] in their review article revised the risk posed by twin gestation against singleton. They conclude that, in terms of risks and cost effectiveness, twin pregnancies represent an entirely reasonable option for IVF patients. They have based their calculations on the idea that correct risk comparisons in a prospective infertility paradigm, therefore, have to compare outcome risks of one twin to* two* consecutive singleton pregnancies and, in addition, should be adjusted for lower outcome risks for IVF twins and higher risks for IVF singletons if obstetrical risk data are utilized in risk comparisons between singleton and twin pregnancies. Though such comparison is beyond the scope of present study, we also believe that twin gestation does avoid additional infertility treatments especially in Indian scenario where two children are often a norm.

### 4.6. Number of Initial Fetuses and Gestational Age/Weight at Delivery

In a prospective observational study it was seen that as the initial number of fetuses increased (in pregnancies reduced to twins) the risk of miscarriage increased and gestational age at delivery decreased [15]. This study had a mean gestational age at delivery of 25.1 weeks in the reduced twin gestation (study group). The number of patients in the study group is very small [15] and it included patients with 5 to 8 fetuses at initial scan. However, similar findings have been reported in the past [4, 6]. Similar trend was seen in present study but the difference was not statistically significant which may be due to exclusion of cases with complete pregnancy loss at 13 weeks.

### 4.7. Birth Weight and Number of Reduced Fetuses

In a large prospective study [5] when singleton deliveries were compared with reduced singleton deliveries, significant advantage was seen in favour of unreduced singleton in terms of both birth weight and term delivery rate. This finding parallels our own. The same study also showed similar findings in twin gestation. Unreduced twins had clinically significantly higher birth weight and lower preterm delivery rate than their reduced counterparts (3 to 2 group). In our own study though we failed to demonstrate a statistically significant advantage, the pattern was similar to the above-mentioned study.

The strength of the present study is that the data was not influenced by performance variability for fetal reduction and nature of treatment. The study also attempts to eliminate bias arising due to maternal indications of pregnancy termination.

Lack of adherent follow-up and retrospective nature of the study are its main limitations. Due to this it was difficult to validate the quality of care delivered especially in patients who were followed up elsewhere.

## 5. Conclusion

Twin pregnancies are at higher risk of lower birth weight and preterm delivery whether they undergo reduction or not. Although reduction does not alter the live birth rate, it does reduce birth weight and gestation age of delivery and the effect is more apparent when multiple gestation is reduced to singleton. Singleton pregnancies fare better than twin pregnancies so ultimate goal of an ART cycle should be the BEST (blastocyst euploid selective transfer).

Our data does not show any statistical difference in the perinatal outcome of reduced versus nonreduced twins; however long term follow-up to study the consequences of reduction is required.

## Figures and Tables

**Table 1:** Perinatal outcome in singleton and twin gestation.

	Singleton		*p* value	Twins		*p* value
	Unreduced	Reduced		Unreduced	Reduced	
Total	50	52		122	68	
Age	31.2 ± 5.7	32.8 ± 6.24	0.1881	32.7 ± 6.4	32.7 ± 6.35	0.983
BMI	23.5 ± 1.2	22.8 ± 2.6	0.0860	22.6 ± 3.5	21.9 ± 1.3	0.114
Primigravida	42	47	0.385	110	64	0.423
Multigravida	8 (16.0)	5 (9.62)		12 (9.83)	4 (5.88)	
Abortion	9 (18)	5 (9.62)	0.259	18 (14.75)	9 (13.23)	0.832
Live birth	41 (82)	47 (90.3)		104 (85.2)	59 (86.7)	
Very preterm delivery	1 (2.4)	4 (8.51)	0.0421	19 (18.27)	11 (16.18)	0.7327
Preterm delivery	2 (4.8)	9 (19.1)		49 (47.1)	25 (42.37)	
Term delivery	38 (92.7)	34 (72.3)		36 (34.6)	23 (33.8)	
Neonatal death	1 (2.4)	2 (4.2)	0.5812	10 (9.61)	5 (8.4)	0.808
IUGR	2 (4.8)	2 (4.2)	0.9681	15 (14.4)	17 (28.8)	0.043

**Table 2:** Outcome in singleton pregnancies (reduced and unreduced).

	Total	3 to 1	2 to 1	1	*p* value
Total	102	6	46	50	
Gestation at delivery	37.44 ± 2.25	36.5 ± 3.27	37.05 ± 2.39	37.98 ± 1.83	0.100
Abortion	14 (13.7)	0	5 (10.9)	9 (18)	0.157
Live birth	88 (86.3)	6 (100)	41 (89.1)	41 (82)	
Very preterm delivery	5 (5.6)	1 (16.7)	3 (7.3)	1 (2.4)	0.030
Preterm delivery	11 (12.5)	0	9 (21.9)	2 (4.8)	
Term delivery	72 (81.8)	5 (83.3)	29 (70.7)	38 (92.6)	
Birth weight	2.79 ± 0.62	2.83 ± 0.75	2.58 ± 0.61	2.99 ± 0.57	0.012

**Table 3:** Outcome in twin gestation.

	Average	4 to 2	3 to 2	2	*p*
Total	190	11	57	122	
Gestation at delivery	35.12 (2.87)	34.30 (3.68)	35.35 (2.6)	35.10 (2.8)	0.572
Abortion	27 (14.2)	1 (9.0)	8 (14.1)	18 (14.7)	0.874
Live birth	166 (85.8)	10 (90.9)	49 (85.9)	104 (85.2)	
Very preterm delivery	30 (18)	4 (40)	7 (14.3)	19 (18.2)	0.847
Preterm delivery	58 (34.9)	2 (20)	23 (46.9)	49 (47.1)	
Term delivery	84 (50.6)	4 (40)	19 (38.7)	36 (34.6)	
Birth weight	1.90 ± 0.56	1.80 ± 0.78	1.85 ± 0.50	1.94 ± 0.57	0.566

**Table 4:** Birth weight of babies born according to gestation and reduction.

	3 to 1	2 to 1	1	4 to 2	3 to 2	2	*p*
Neonates born	6	41	41	19	95	192	
ELBW (<999 gm.)	0	0	0	3 (15.7)	1 (1.1)	5 (2.6)	<0.0001
VLBW (1 to 1.5 kg)	2 (33.3)	2 (4.8)	1 (2.4)	5 (26.3)	21 (22.1)	43 (23.3)	
LBW (1.6 to 2.5 kg)	0	16 (39.2)	16 (39.2)	8 (42.1)	67 (70.5)	127 (66.1)	
NBW (>2.6 kg)	4 (66.7)	23 (56.0)	24 (58.4)	3 (15.7)	6 (6.3)	17 (8.8)	
Average	2.83 ± 0.75	2.58 ± 0.61	2.99 ± 0.57	1.80 ± 0.78	1.85 ± 0.50	1.94 ± 0.57	

## References

[1] Sciarra J. (1994). Infertility: an international health problem. *International Journal of Gynecology and Obstetrics*.

[2] World Health Organization (2004). *Infecundity, Infertility, and Childlessness in Developing Countries*.

[3] Serour G. I. (2006). Ethical recommendations on multiple pregnancy and multifetal reduction: FIGO Committee for the Ethical Aspects of Human Reproduction and Women's Health. *International Journal of Gynecology & Obstetrics*.

[4] Hershko-Klement A., Lipitz S., Wiser A., Berkovitz A. (2013). Reduced versus nonreduced twin pregnancies: obstetric performance in a cohort of interventional conceptions. *Fertility and Sterility*.

[5] Zhang Y.-L., Wang X.-Y., Wang F., Su Y.-C., Sun Y.-P. (2015). Clinical analysis of spontaneous pregnancy reduction in the patients with multiple pregnancies undergoing in vitro fertilization/intracytoplasmic sperm injection-embryo transfer. *International Journal of Clinical and Experimental Medicine*.

[6] Cheang C.-U., Huang L.-S., Lee T.-H., Liu C.-H., Shih Y.-T., Lee M.-S. (2007). A comparison of the outcomes between twin and reduced twin pregnancies produced through assisted reproduction. *Fertility and Sterility*.

[7] Kandraju H., Agrawal S., Geetha K., Sujatha L., Subramanian S., Murki S. (2012). Gestational age-specific centile charts for anthropometry at birth for south Indian infants. *Indian Pediatrics*.

[8] Berkovitz A., Hershko-Klement A., Fejgin M. (2010). Nulliparity, fertility treatments and twins: a time for rethinking. *Fertility and Sterility*.

[9] Haas J., Mohr Sasson A., Barzilay E. (2015). Perinatal outcome after fetal reduction from twin to singleton: to reduce or not to reduce?. *Fertility and Sterility*.

[10] Helmerhorst F. M., Perquin D. A. M., Donker D., Keirse M. J. N. C. (2004). Perinatal outcome of singletons and twins after assisted conception: a systematic review of controlled studies. *The British Medical Journal*.

[11] Yaron Y., Bryant-Greenwood P. K., Dave N. (1999). Multifetal pregnancy reductions of triplets to twins: comparison with nonreduced triplets and twins. *American Journal of Obstetrics and Gynecology*.

[12] Depp R., Macones G. A., Rosenn M. F. (1996). Multifetal pregnancy reduction: evaluation of fetal growth in the remaining twins. *American Journal of Obstetrics and Gynecology*.

[13] Gleicher N., Bard D. H. (2013). Mistaken advocacy against twin pregnancies following IVF. *Journal of Assisted Reproduction and Genetics*.

[14] AlShelaly U. E., Al-Mousa N. H., Kurdi W. I. (2015). Obstetric outcomes in reduced and non-reduced twin pregnancies. A single hospital experience. *Saudi Medical Journal*.

[15] Alexander J. M., Hammond K. R., Steinkampf M. P. (1995). Multifetal reduction of high-order multiple pregnancy: comparison of obstetrical outcome with nonreduced twin gestations. *Fertility and Sterility*.

